# Diagnosing Pain in Individuals with Intellectual and Developmental Disabilities: Current State and Novel Technological Solutions

**DOI:** 10.3390/diagnostics13030401

**Published:** 2023-01-22

**Authors:** Meir Lotan, Michal Icht

**Affiliations:** 1 Department of Physical Therapy, Ariel University, Ariel 40700, Israel; 2 Israeli Rett Syndrome Association, National Evaluation Team, National Rett Syndrome Clinic, Chaim Sheba Medical Center, Ramat-Gan 526200, Israel; 3 Department of Communication Disorders, Ariel University, Ariel 40700, Israel

**Keywords:** pain assessment, pain management, intellectual and developmental disabilities

## Abstract

Pain assessment poses a challenge in several groups of clients, yet specific barriers arise when it comes to pain assessment of individuals with intellectual and developmental disabilities (IDD), due mostly to communication challenges preventing valid and reliable self-reports. Despite increased interest in pain assessment of those diagnosed with IDD within recent years, little is known about pain behavior in this group. The present article overviews the current state of pain diagnosis for individuals with IDD, focusing on existing pain assessment scales. In addition, it suggests technological developments offering new ways to diagnose existence of pain in this population, such as a Smartphone App for caregivers based on unique acoustic characteristics of pain-related vocal responses, or the use of smart wearable shirts that enable continuous surveillance of vital physiological signs. Such novel technological solutions may improve diagnosis of pain in the IDD population, as well as in other individuals with complex communication needs, to provide better pain treatment and enhance overall quality of life.

## 1. Introduction

The existing literature suggests that pain in individuals with severe IDD is a common phenomenon, yet at the same time is rarely actively treated [1]. Individuals with IDD suffer from health problems at a rate 2.5 times higher than that of the neurotypical population [2]. Moreover, those diagnosed at severe to profound levels of IDD are more likely to have multiple complex medical problems, which may co-occur with communication difficulties. These disabling conditions often necessitate painful medical procedures (e.g., medical interventions, physical therapy treatments). Finding ways to overcome communication obstacles in the assessment of pain in individuals with IDD may result in more timely and effective treatment of pain not just for these individuals but also for other populations that may not be able to verbally communicate pain, such as very young children, individuals with autism spectrum disorder (ASD), and very old adults. Thus, this article overviews the current state of pain diagnosis for individuals with IDD and suggests technological developments offering new ways to assess pain in this population.

The International Association for the Study of Pain (IASP) defines pain as “an unpleasant sensory or emotional experience associated with actual or potential tissue damage or described in terms of such damage” [3]. Pain is usually accompanied by adverse effects, negatively influencing one’s mobility, functional ability, work capability, interpersonal relationships, social activities, and emotional state. In turn, these may lead to increased use of health care services and higher health care costs [4]. In addition, as the IASP notes: “The inability to communicate verbally does not negate the possibility that an individual is experiencing pain and is in need of appropriate pain-relieving treatment”. It is, therefore, crucial to develop pain assessment methods to address the discomfort of individuals suffering from pain which cannot be voiced. Failure to successfully and quickly assess pain is viewed nowadays as poorly applied medicine, unethical practice, and an annulment of fundamental human rights [5].

In addition to the aforementioned medical and functional issues, individuals with IDD are vulnerable to pain inflicted by poorly fitted wheelchairs and leg braces [6]. While pain evaluation is extremely problematic, mostly due to communication difficulties, some studies have attempted to estimate the prevalence of pain experience by individuals with IDD [7]. One such research investigation examined the duration, frequency, location and intensity of pain, as well as the occurrence of pain during daily activities in individuals with cerebral palsy (CP) and IDD, and suggested that pain was a substantial problem for most participants [8]. Chronic pain (defined by the researchers as persisting for a minimum of three months) was reported in 67% of the participants, while 53% experienced moderate to severe pain almost every day. Most complaints were reported as back (63%) and lower-extremity pain (66%). Upper-extremity pain was experienced for a mean of 7.5 years while hip or buttock pain had a duration of up to 20 years. Similar results were reported by Breau and associates [9], who assessed pain in 94 youngsters aged 3–18 years with various levels of IDD, ranging between moderate to profound. Seventy-three participants (78%) were reported as suffering from pain at least once, while 35% to 52% had pain on a weekly basis. Musculoskeletal or gastrointestinal problems were the most common problems, causing, on average, about nine weekly hours of pain [9]. In people with severe/profound IDD, who usually lack communication abilities, there is a greater likelihood of experiencing (chronic) pain in the long term [9].

Taken together, these reports suggest that individuals with IDD at all ages are suffering from more pain than their neurotypical peers. Additional inquiries are called for to examine how pain management can be improved for individuals with IDD and multiple disabilities [8]. Clearly, appropriate pain assessment and diagnosis are the initial crucial steps to improving pain management. 

## 2. Diagnosing Pain in Individuals with IDD

Pain assessment in care-receivers with IDD is a complex task and can become extremely challenging, especially for those with IDD diagnosed at the severe and profound levels, as their ability to verbally communicate their pain experience is seriously compromised [10]. Consequently, objective assessment tools are essential: Without them, pain sensation may be misinterpreted or underestimated by caregivers, possibly leading to deficient pain administration, thereby undermining the quality of life (QoL) of this vulnerable group [11]. In light of the negative implications on the QoL, there is a critical need to develop new measurement devices, such as auditory methods, or pain assessment tools for this group of clients. However, the academia has lagged behind in developing methods to assess pain for individuals with IDD. This situation can be explained in large part by the complexity of pain diagnosis in this group, as described below. 

### 2.1. Complex Diagnosis of Pain in Individuals with IDD

Pain diagnosis in people with IDD is challenging due to multiple factors. Firstly, people with IDD typically have neurological issues that may influence their ability to effectively communicate their pain experience, thus complicating its evaluation [12]. For example, they may face difficulties in responding to questions regarding their pain or may react in a way that is not conventional and therefore may not be meaningful to their caregivers [13]. Cognitive impairments in abstract thinking and spatial orientation (misunderstanding their body scheme) are also common in these individuals. Such communication and cognitive deficits may lead to difficulties in reliable reports of the specific elements constructing their pain sensation, such as intensity, position, and/or quality. These impediments make pain measurement in this group of service receivers extremely complicated or even impossible [14]. Self-report is considered the ‘gold standard’ of pain assessment. Yet such a measure is extremely challenging to implement when it comes to care-receivers with neurological difficulties. 

Secondly, individuals with IDD often have a wide range of possible handicaps and limitations, making them a heterogeneous group in terms of function, communication, and behavior. Thirdly, their pain expression might be masked due to limited movement (in cases of paralysis) [15] or challenging behaviors common in the IDD population, such as self-injury, aggression, and tantrums [16]: it is difficult to determine whether such these are ordinary behaviors of the individual or might be caused by painful medical problems [17,18,19] or some other source of distress. Moreover, prevalent behavioral pain markers within the general population, such as moaning, specific facial expressions and altered sleep patterns [20], presented by care-receivers with IDD during times of no pain [15] and thus may be attributed to the intellectual level of the person with IDD rather than to the existence of pain, leading to misdiagnosis [21]. 

### 2.2. Existing Pain Scales

Despite the enhanced research focus on pain-related expressive behavior in people with IDD [22,23,24,25,26], scientific investigation regarding this topic is limited, and there are but few available pain-assessment scales constructed specifically for this group of people. Most have been introduced in recent years, and, of them, the majority are for children (marked with ^#^ in the Table A1 in Appendix A). The following scales are presented in a chronological order, and their main psychometric values and features are reviewed in Appendix A.

(1) The Facial Action Coding System (FACS) [27]: The FACS provides a list of muscular facial action units (AUs), based on specific facial muscle movements that may occur separately or in combination (as groups). FACS was found time and again to be a highly reliable scale for neurotypical adults by Craig and associates [28,29,30,31], as well as by other researchers [32]. FACS has been used in adults with dementia [33,34], and in individuals with mild to moderate levels of IDD undergoing seasonal influenza injections [11]. A pediatric adjustment of the Child Facial Coding System (CFCS; [35]) has been implemented for pain assessment in postoperative situations in a pediatric population with IDD [36]. Nevertheless, the FACS and CFCS are relatively user-complicated and, while they have been found to be appropriate for research purposes, their use in clinical settings is not yet feasible. 

(2) The Evaluation Scale for Pain in Cerebral Palsy (ESPCP) [37]: The ESPCP is based on cues indicative of pain according to physicians’ reports, and consists of 22 items. The items denote behavioral expressions, such as body movements, crying, and postural changes (e.g., increase in muscular tone, analgesic postures, involuntary movements); protective reactions (e.g., covering suspected painful areas), and social behaviors (e.g., reduced interest in people and physical surroundings). This set of behaviors was found indicative of pain experience of care-receivers at a severe level of IDD. The authors of the scale report that an array of different items was found to differ between clients in determining pain and was related to the personal level of development and cognitive ability of each participant. 

Collignon et al. [38] developed an observational pain scale of ten items of the ESPCP, for the pediatric population with severe handicaps as well as for adults with CP. The scale was further developed to adhere to adolescents with IDD [39]. The researchers evaluated 100 individuals between the ages of 2–33 years (mean: 16 years), showing an array of disabilities, profound level IDD, and a lack of verbal as well as symbolic communication. The authors acknowledged that pain existence of these care-receivers could only be identified by monitoring an array of behavioral changes, and could not be based on a specific sign. In addition, the researchers reported that various combinations of disabilities were found to elicit distinct behaviors. For instance, the researchers found that a lower degree of motor impairment was associated with an increase in voluntary protection of painful areas. No further investigation of the psychometric properties of the ESPCP has been conducted. Additional research is required to establish this scale as a psychometrically valid and reliable clinical tool. 

(3) The Non-Communicating Children’s Pain Checklist (NCCPC ^#^) [40]: At the initial stages of this tool’s development, McGrath and associates [15] interviewed 20 care-providers (including parents) of children and young adults diagnosed at a severe level of IDD (age range of 6–29 years). During interviews held by the researchers, a set of pain-indicative cues was collected. The interviews focused both on occurrences of short, sharp pain (e.g., needle-like pain), as well as incidents of longer-lasting pain (e.g., headache or long-lasting pain resulting from a physical injury). This inquiry elicited a list of 30 cues. Though specific behaviors differed among the care-receivers (as reported by their caregivers) [38,39], groups of behaviors (e.g., vocal, social/personality, body-and-limb activity, facial expressions, eating/sleeping, and physiological reactions) were common to many of the participants. Following this initial inquiry, the NCCPC, consisting of 30 items, was introduced [40].

At this point, the NCCPC was implemented by parents and other care-providers within in a home setting. The evaluation of the 30 pain behaviors took place in four different daily situations: a calm non-painful situation, a distressing non-painful situation, acute pain, and long-term pain. Findings indicated that painful situations were characterized by more than four times as many pain cues as non-painful situations. 

The NCCPC-PV (PV = Postoperative Version) is a second version of the original NCCPC, assessing pain behaviors in a post-operative situation [41]. In this study, items related to eating and sleeping were omitted, and the remaining items were then scored on a four-point scale, according to occurrence. A care-provider and a researcher observed 24 children, (age range: 3–19 years), in pre- and post-surgery settings. Each of the two observers rated the intensity of the child’s pain using a Visual Analogue Scale (VAS), and completed the NCCPC-PV (independently). This new version of the scale (NCCPC-PV) was found to have high psychometric values—e.g., very high internal consistency (Cronbach’s alpha = 0.91) and good inter-rater reliability (ICC 0.78 to 0.82). 

The NCCPC-R (R = revised) is a third version of this scale. Previously omitted items, such as items related to food consumption or sleep duration and quality, are included in this scale. The NCCPC-R was tested at home settings [42], where care-providers observed their child during painful and non-painful periods. Seventy-one children, between the ages of 3 to 18 years, diagnosed with severe IDD, participated in the study. The scale had high internal consistency (Cronbach’s alpha = 0.93), and a moderate correlation with the pain intensity ratings provided by care-providers (Pearson’s *r* = 0.46). Specificity and sensitivity for pain (0.77 and0.84, respectively) were optimized at a cut-off point of 7 out of a possible total score of 90. Two other pain scales are also based on the NCCPC-R: (a) The Batten’s Observational Pain Scale (BOPS), developed for those diagnosed with Neuronal Ceroid Lupofiscinosis on top of their IDD diagnosis [43], and (b) the Chronic Pain Scale for Nonverbal Adults with Intellectual Disabilities (CPS-NAID) [44].

(4) The Pain Indicator for Communicatively Impaired Children (PICIC ^#^) [26]: This tool evaluates the manifestation of chronic pain in a pediatric population, using six items to assess experience in non-verbal participants with IDD at severe/profound levels. Significant association was found between five of the six items and the occurrence of pain as well as its severity [26]. This scale is not yet considered psychometrically sound and requires more study. 

(5) The Pediatric Pain Profile (PPP ^#^) [45]: The PPP is a rating scale consisting of 20 behavioral items for the assessment of pain in children with significant cognitive and neurological disabilities. The reliability and validity of the scale were assessed in a group of 140 children with complex communication needs, aged 1–18 years, who were unable to communicate either verbally or through augmentative communication devices. The scale was used by parents to retrospectively evaluate their children’s behavior in no-pain situations and painful situations. The children were found to display significantly higher scores in a painful situation than in a no-pain situation. Inter-rater reliability (using an Intraclass Correlation Coefficient) ranged from 0.74 to 0.89. Behaviors of 41 children were ranked before the administration of an analgesic and four hours afterwards, to assess the construct validity and responsiveness of the scale. A significant difference was found between the PPP scores pre- and post- analgesic treatment. Internal consistency (Cronbach’s alpha) ranged from 0.75 to 0.89, and sensitivity and specificity (1.00 and 0.91, respectively) were optimized at a cut-off point of 14 on the 60-point scale. The highest PPP score was recorded in the first 24 h post-surgery in 14 of 30 children (47%), for whom scores were also collected pre-surgery, and up to 5 days after surgery (no significant difference was found between pre-operative and post-operative scores). 

The PPP needs to demonstrate more rigorous psychometric properties to qualify as a psychometric scale with acceptable values. Further research is necessary to assess the usefulness, feasibility, and acceptability of the PPP as a tool in clinical settings for a pediatric population with severe to profound cognitive disabilities. It is yet to be concluded whether this scale may also be suitable for assessment of pain in an adult population with comparable levels of disability [45].

(6) The Pain and Discomfort Scale (PADS) [20]: The PADS is based on the NCCPC-R [41,42] scale and was developed for use in the course of a physical examination. According to the developers, training is required by the scale-users before it can be use in clinical settings or research. As part of the development of the PADS, Bodfish et al. [20] conducted a series of three studies, in which all participants presented significant reductions in PADS scores from pre-treatment state to treatment, thus this scale was found indicative of pain reduction [20]. The three studies were conducted as follows:

(a) Twenty-two adults diagnosed at a severe or profound IDD level were evaluated for pain behaviors presented before and during acute medical procedures assumed to produce pain and/or discomfort (procedures such as a toenail removal or a gastronomy-tube insertion).
(b) The pain scores of a group of individuals with physical disabilities and painful chronic medical conditions were matched to the pain scores of a group with IDD alone (no-pain group; the scores of the former were found to be significantly higher); and
(c) Eight adults diagnosed at a profound level of IDD with various concurrent medical conditions were assessed before and after pain-killer use.

The PADS [20] was later used during a dental scaling procedure to detect discomfort and pain [46]. Twenty-eight participants with communication and cognitive challenges were evaluated across multiple baselines, in addition to on-going evaluations during and after the procedure. The authors found that the PADS scores significantly rose during the procedure. The PADS still lacks a cut-off point for sensitivity and specificity [46]. Existing scientific evidence supports the fact that this scale can be utilized as a measurement of pain in adults with IDD [20,46]. 

(7) The Non-Communicating Adult’s Pain Checklist (NCAPC): This tool is based on earlier pediatric research regarding body movements and facial expressions as indicators of discomfort and acute pain [41,42]. In the preliminary stages of the development project [47], the behaviors of 121 individuals with IDD and a comparison group of 38 individuals with normal cognition were evaluated by two independent raters, using the FACS [27] and the NCCPC-R [41,42]. Monitoring behaviors before and during influenza vaccination, the study indicated that both baseline and pain behavior were influenced by cognition. In addition, it was found that the FACS might provide a false impression that people with severe and/or profound IDD are less sensitive to pain stimuli. Finally, in this sample, the NCCPC-R was found to be a more sensitive pain measure than FACS. 

In a later study [48], 228 adults with different levels of IDD were video-recorded before and during an influenza vaccination, and their pain reactions were scored using the NCCPC-R. Each of the 30 items on the original scale was examined for sensitivity to change (from baseline to a painful situation) and internal consistency; in addition, sensitivity to change in the total scale as well as of subscales was examined. This systematic procedure yielded a novel scale: the Non-Communicating Adults Pain Checklist (NCAPC). The NCAPC was then re-scored using a random sub-sample of 89 participants, and all items were found to be sensitive to pain, with satisfactory internal consistency (α = 0.77), and high sensitivity to pain for individuals at all levels of IDD (SRM ranging between 1.20 and 2.07). The findings suggest that NCAPC provides improved psychometric measures of acute pain than does that NCCPC-R in this sample of adults. In another study, caregivers, and physical/occupational therapists were evaluated for inter-rater reliability (ICC) of the NCAPC, which was 0.92 and 0.91, respectively [49].

In another study, 58 adults with IDD at various cognitive and functional levels were observed before and during a dental hygiene treatment (characterized by high pain levels) and while taking an influenza vaccination (lower pain level). The results showed that the NCAPC was sensitive enough to distinguish between pain and non-pain conditions, as well as between high and low levels of pain occurrences. Notably, the scale was found to be sensitive in detecting pain at all levels of IDD. Moreover, the fact that the data were collected in a clinical setting (not recorded on video) supported the clinical utility of the NCAPC [50]. A pain model was constructed with scores collected using the NCAPC [51], further confirming the construct validity of the set of pain cues used within the scale.

Even though some of the above-mentioned studies were performed in clinical settings, there are no publications describing the use of any of these scales in such settings on a regular basis. Therefore, the extent to which these scales are being clinically used is unclear. For some of the extensively studied scales, particularly the various versions of the NCCPC as well as the NCAPC, psychometrics evidence and feasibility are available. Yet, for most pain assessment scales, clinical feasibility studies are still warranted at this point. Clinical use of pain assessment scales is required to extend them beyond the academic field, to aid professionals and, ultimately, to allow for more effective pain management for this group of clients. One of the major disadvantages of using observational scales is the fact that these scales are based on proxy reports which were found, in some cases, to reflect disagreement between the observed person and his proxy [52]. This predicament suggests the use of more objective potions such as the ones using physiological measures. 

### 2.3. Physiological Pain Assessment

In addition to the abovementioned scales for pain assessment in individuals with IDD, there are a number of physiological measures of pain in use, including vagal tone [53], heart rate [54], blood pressure [55], salivary amylase activity [56], respiration [57], and intracranial pressure [58]. Although physiological measures may be viewed as free of response bias and therefore more conducive to objectivity, no single physiological index has been shown to be ideal and specific enough for measuring pain. 

In the past, physiological measures were impractical in terms of the time and costs associated with their use, especially when considering the existing conditions in institutions for individuals with IDD. Yet, due to current developments in technology, physiological measures might be considered for use as neutral/objective indicators to assess pain in individuals with IDD. In light of such technological developments, the next section will suggest the application of existing tools that might enable the development of physiologically dependent pain assessment possibilities for individuals with IDD. 

## 3. New Technological Solutions Enabling Pain Assessment in Individuals with IDD

### 3.1. Assessing Pain Using Vocal Responses

Technology may assist in pain management for chronic pain patients through various chronic pain apps and pain trackers (e.g., CatchMyPain^®^ App, My Pain Diary^®^ App). Such pain apps enable patients to easily track episodes of chronic pain, locate their pain on their body, and track potentially related factors, such as stress, fatigue, mood and even weather. However, such apps are based on self-report, and thus are not suitable for the IDD population. 

Aiming to overcome this barrier, other apps, such as the PainChek^®^ App (PainChek Limited, Sydney, Australia), employ AI facial recognition technology. Typically, a smartphone camera directed at the patient’s face is used to record facial muscle movements indicative of pain, and the app calculates an overall pain score. Yet, as aforementioned, such behavioral pain indicators may be detected in people with IDD when they do not suffer from pain [15], leading to misdiagnosis. 

A possible solution may be based on pain-related vocal responses, such as non-verbal cries, moans or screams, which are produced by individuals experiencing pain [58,59]. For those who are unable to adequately self-report their subjective pain experience, such as older adults with advanced dementia [60,61], vocalizations may be used as indicators of pain. Indeed, some researchers pointed to the ability to evaluate pain in non-verbal individuals with IDD using their vocal expressions [15,22]. 

**Figure 1:**
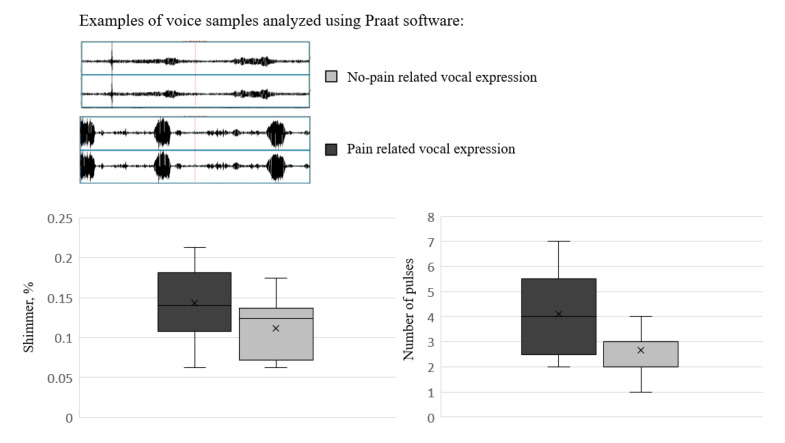
Results of Icht et al., 2021 [62]: Examples of the acoustic analysis of the voice recordings for pain- and no-pain related vocal expressions, and Box plots depicting the main findings: Shimmer level (%, the left panel bar plot), and number of pulses (the right panel bar plot).

In a recent preliminary study, the authors [62] indicated using acoustic features of vocal expressions to identify pain in adults with IDD. Nine adults with a severe-profound IDD level were recorded in daily activities associated with pain (during diaper changes), or no-pain (at rest). Acoustic analyses of spontaneous vocal expressions revealed that pain-related vocalizations were characterized by a higher number of pulses (i.e., segments in which phonation was detected), and higher shimmer values (the cycle-to-cycle amplitude variation) relative to no-pain vocal expressions (for a visual illustration of these results, see Figure 1). These pain-related vocal characteristics may be used to develop objective pain detection means, such as a smartphone app for caregivers. 

### 3.2. Using Smart Shirts as a Base for Pain Assessment 

As mentioned above, physiological data associated in the past with pain reaction are heart rate [53], respiration [56] as well as other physiological measures. Smart wearable shirts (SWS) are wearable medical devices that are considered a technological breakthrough, enabling continuous surveillance of human vital physiological signs without any disturbance to the activities of daily living. The SWS technology has been used in clinical research for the past two decades. During the past few years, SWS has enabled the collection of varied physiological data outside the laboratory (for weeks at a time).

The constant surveillance enabled by these devices allows for the identification of physiological anomalies that deviate from the typical individual’s behavior; data on the anomalies can be received and analyzed, enabling treatment [62]. Furthermore, garments such as t-shirts, have been found to be the second most preferred device to be used by individuals with ASD [63], with a moderate to high suitability index for this population [64,65]. Existing SWS can collect physiological data including heart rate, respiratory function and change in regular respiration, body movements and pacing, and therefore might be used as data collecting devices detecting pain signs of individuals with IDD. Other accessories or wearable sensors, such as smart electronic bracelets, may be used for this purpose as well [66,67].

## 4. Conclusions

Over the past 20 years, developments have been made in understanding the pain experience of individuals with IDD. Yet much remains unknown. Scientific findings suggest that individuals with IDD suffer more pain than neurotypical individuals, and that their pain is under-managed. This may lead to reduced function for the individual [68], challenging behavior [16,19,69], and much suffering. Painful situations are a significant cause of concern for both parents and caregivers [22], as well as a source of frustration for healthcare professionals [25]. Moreover, pain-causing medical issues are a common cause for hospital admissions [70], resulting in a burden on both persons with IDD and their direct care-providers. 

We know that pain is a complicated as well as subjective phenomenon, two elements making its diagnosis difficult, particularly when it comes to individuals presenting severe communication difficulties such as those common in individuals with IDD. Scientific findings suggest that even when the phenomenon of pain is assessed in individuals with IDD, it is many times under-treated when compared to other populations [71,72]. 

Leading specialists in this field of research believe that most of the pain treatments available for neurotypical care-receivers can undoubtedly be used in those with the IDD diagnosis. Such pain treatments should be delivered in an appropriate manner, adapted to their cognitive abilities and health status, and in accordance with physical limitations, current medication consumption, and coexisting health conditions. 

For this special group, it is highly recommended to provide multidisciplinary care, so that multiple therapies can work synergistically, and so that professionals can receive support. Good pain management for this group of care-receivers should be considered a high-priority element administered as a part of a comprehensive health management and habilitation program. 

Due to the commonality of pain among individuals with IDD, we urge that pain be monitored regularly through the use of appropriate and validated diagnostic tools, thereby enabling proper pain management. This may require support of care-providers in collecting pain ratings at home, perhaps with the support of new technological devices. Such measures are needed since those with IDD may not show typical or familiar signs of pain during medical procedures. 

Therefore, it is important to evaluate pain in this group of clients in a broad context, considering a variety of factors such as pain perception and behavior of the individuals, their environment and previous experience, as well as ongoing development, as all these factors may influence their external pain presentation [11]. Frameworks such as the International Classification of Functioning, Disability and Health may be helpful in this important task [73]. Constant development of technology in all areas may come to the aid of individuals with IDD, and areas which until lately were under researched (such as physiological measures, and audio-based applications), may now become the up-coming pain-assessment-tools easing the detection of pain in this group of clients over-coming the existing barriers in detecting pain in this group of service-receivers.

## Appendix A

**Table A1:** A comparative summary of existing pain scales for individuals with IDD.

Name	FACS *^#^	ESPCP ^#^	NCCPC ^#^	PICIC ^#^	PPP ^#^	PADS *	NCAPC *
No. of items	8	22; 10	30	6	20	18	18
Source	Lachapelle et al., 1999 [10]	Giusiano et al., 1995 [37]; Collignon et al., 1997 [38]	McGrath et al., 1998 [15]; Breau et al., 2000 [40]; Breau et al., 2002 [42]	Stallard et al., 2001 [1]; Stallard et al., 2002 [26]	Hunt et al., 2004 [45]	Bodfish et al., 2001 [20]; Phan et al., 2005 [46]	Lotan et al., 2009 [49]; Lotan et al., 2010 [50]
Population	Adults with mild-moderate IDD	Adolescents with CP & IDD	Children with cognitive impairment	Non communicating children	Children with severe neurological and cognitive disability	Adults with severe and profound MR	Adults at all levels of IDD
Age range (mean)	(49.6)	2–33 (16)	6–29 (14.5); 3–9; 3–18	2 + (9.4); 2–21 (7.8)	1–18 (9.9)	-----	
N	40	100	20; 24; 71	49; 34	140	22; 8	228
Type of pain	Procedural	Not specified	Needle pain; long lasting pain	Non specific—severe pain	Not specified	Procedural; Chronic	Procedural; Chronic
Psycho-metric properties:	Have not been tested	Have not been tested	0.78–0.82	Have not been tested	0.74–0.89	0.86	0.94–091
(a) Reliability							
(b) Internal consistency			0.91–0.93		0.75–0.89	-----	0.77
(c) Validity			-----		Construct validity	Content validity	Content validity Construct validity
(d) Specificity			0.81–0.74		1.00	-----	-----
(e) Sensitivity			0.88–0.84		0.91	-----	-----

Index—Scales validated for adults with IDD *; Scales validated for children with IDD ^#^; Scales validated for children and adults with IDD *^#.^
